# Epidemiology, surveillance and diagnosis of Usutu virus infection in the EU/EEA, 2012 to 2021

**DOI:** 10.2807/1560-7917.ES.2023.28.33.2200929

**Published:** 2023-08-17

**Authors:** Giorgia Angeloni, Michela Bertola, Elena Lazzaro, Matteo Morini, Giulia Masi, Alessandro Sinigaglia, Marta Trevisan, Céline M. Gossner, Joana M. Haussig, Tamas Bakonyi, Gioia Capelli, Luisa Barzon

**Affiliations:** 1 Istituto Zooprofilattico Sperimentale delle Venezie, Viale dell’Università 10, Legnaro (Padua), Italy; 2 Department of Molecular Medicine, University of Padua, Via Gabelli 63, Padua (Padua), Italy; 3 European Centre for Disease Prevention and Control (ECDC), Solna, Sweden; * These authors contributed equally to the work and share first authorship.; ** These authors contributed equally to the work and share last authorship

**Keywords:** Usutu virus, surveillance, diagnosis, Europe, One Health

## Abstract

**Background:**

Usutu virus (USUV) is a flavivirus with an enzootic cycle between birds and mosquitoes; humans are incidental dead-end hosts. In Europe, the virus was first detected in Italy in 1996; since then, it has spread to many European countries.

**Aim:**

We aimed to report on the epidemiology, surveillance, diagnosis and prevention of USUV infection in humans, mosquitoes and other animals in the European Union/European Economic Area (EU/EEA) from 2012 to 2021.

**Methods:**

We collected information through a literature review, an online survey and an expert meeting.

**Results:**

Eight countries reported USUV infection in humans (105 cases, including 11 with neurological symptoms), 15 countries in birds and seven in mosquitoes. Infected animals were also found among pets, wild and zoo animals. Usutu virus was detected primarily in *Culex pipiens* but also in six other mosquito species. Detection of USUV infection in humans is notifiable only in Italy, where it is under surveillance since 2017 and now integrated with surveillance in animals in a One Health approach. Several countries include USUV infection in the differential diagnosis of viral encephalitis and arbovirus infections. Animal USUV infection is not notifiable in any EU/EEA country.

**Conclusion:**

Human USUV infections, mainly asymptomatic and, less frequently, with a febrile illness or a neuroinvasive disease, have been reported in several EU/EEA countries, where the virus is endemic. Climate and environmental changes are expected to affect the epidemiology of USUV. A One Health approach could improve the monitoring of its evolution in Europe.

**Key public health message**

**What did you want to address in this study?**

Usutu virus (USUV) is widespread in Europe, where it circulates between birds and mosquitoes. Its impact for human health should be better assessed. We collected information on USUV epidemiology, surveillance, diagnosis and preventive measures through literature review, an online survey and an expert meeting.

**What have we learnt from this study?**

From 2012 to 2021, ca 100 human cases of USUV infection were reported in several EU/EEA countries where the virus is present. Most of those infected had no or only mild symptoms (fever) and rarely neurological involvement. Usutu virus had less public health impact than West Nile virus, which caused outbreaks of encephalitis during the same period. Infection with USUV was also detected in several mosquito and bird species, as well as in horses and other mammals.

**What are the implications of your findings for public health?**

To date, USUV is not a major public health threat in the EU/EEA but monitoring virus circulation and its pathogenicity is important to early detect any change in the epidemiology of the disease. Improvements in monitoring might be achieved with a common case definition of USUV infection in the EU/EEA, and with an integrated approach, including humans, animals and vectors in USUV surveillance.

## Introduction

Usutu virus (USUV) is a flavivirus of African origin transmitted mainly by mosquitoes of *Culex* species (spp.) to multiple bird species, which act as amplifying hosts. In Europe, this virus was first detected in 1996 in Italy, where it caused an outbreak among birds [1]. Conceivably, the virus spread from Italy to the neighbouring countries and is now present in many western, southern and central European countries, where it has been detected in humans, vertebrate hosts and/or mosquitoes [2]. Phylogenetic analyses grouped USUV strains into eight distinct lineages, i.e. Africa 1–3 and Europe 1–5, of which Europe 2 lineage is the most commonly detected in European countries [2]. Usutu virus often co-circulates with West Nile virus (WNV) in a potentially overlapping geographic and ecological range (i.e. avian hosts and mosquitoes), sharing the same environment [3].

Usutu virus causes mortality in several wild bird species, mainly in Eurasian blackbird (*Turdus merula*) and great grey owl (*Strix nebulosa*) [4,5]. Infected mosquitoes can transmit the virus to humans and other mammals, which represent dead-end hosts. Direct human-to-human transmission may potentially occur through infectious substances of human origin (SoHO) [6].

Most USUV infections in humans are asymptomatic and most notified infections have been incidentally detected through screening of asymptomatic blood donors by WNV nucleic acid amplification tests (NATs) that cross-react with USUV [6,7]. Notably, in some areas, asymptomatic USUV infections may be more common than WNV infections, as documented by longitudinal testing of blood donors in the Netherlands [8] or by serosurveys in Italy and France [7,9,10]. A few cases of symptomatic USUV infections have been reported in immunocompromised and/or elderly individuals with neurological symptoms [11-16] or with febrile illness [15].

Laboratory diagnosis of USUV infection in humans is based on demonstration of a specific antibody response against the virus, detection of viral RNA and virus isolation in cell culture from body fluids. The presence of USUV RNA has been demonstrated in blood, urine and cerebrospinal fluid (CSF) of patients with acute infection [13-15]. Diagnosing USUV infection in humans is challenging because of the cross-reactivity of antibodies with other related flaviviruses, such as WNV. Thus, neutralisation assays against both viruses need to be run in parallel for confirmation [15,17].

Knowledge on the epidemiology and impact of USUV infection on human health would be helpful for public health authorities to decide on the possible implementation of surveillance and control measures. Here, we provide an update on the following aspects of USUV in the EU/EEA countries: (i) the epidemiological situation in humans and animals; (ii) the surveillance systems in place; (iii) the diagnostic capability and the main methods applied for laboratory diagnosis and (iv) the preventive measures for the safety of SoHO supply.

## Methods

Data and information on USUV epidemiology, surveillance and diagnosis in 30 EU/EEA during the period 2012 to 2021 were collected through:

(i) a review of peer-reviewed articles reporting USUV detection in humans, vertebrate animals and mosquitoes from 2012 to 2021 using five search engines and platforms (PubMed, Web of Science, Scopus, Embase and CAB-abstracts);

(ii) an online survey distributed to the European Centre for Disease Prevention and Control (ECDC) National Focal Points (NFP) for Emerging and Vector-borne Diseases (EVDs), European Food Safety Authority (EFSA) NFP and SoHO national competent authorities in the period from July to September 2021. The ECDC NFPs are official representatives of the national public health institutes and the EFSA NFPs are official representatives of the food safety and/or the veterinary authorities or institutes in the EU/EEA countries. The EFSA NFPs were requested to forward the survey to the National Competent Veterinary Authority.

(iii) a technical stakeholder meeting involving representatives from national veterinary and public health institutes and SoHO authorities in addition to invited scientific experts. The aim of the meeting was to share and clarify the information collected though the questionnaire.

Although UK was an EU/EEA country until the end of 2020, this country was not included in the online survey and the technical stakeholder meeting, which were conducted in 2021–2022.

More details on the methods and the research strings used in the review are available in the Supplement.

## Results

### Epidemiological situation

Data on USUV in humans, mosquitoes and other animals derived from the survey and the peer-review literature were compared and merged in order to have the most complete information.

#### Human infections

**Figure 1:**
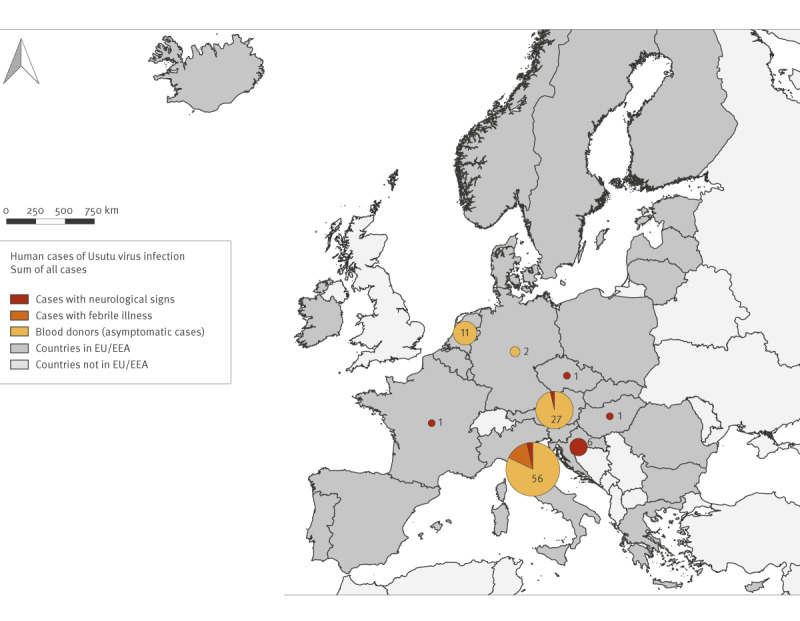
Cases of Usutu virus infection in humans, EU/EEA countries, 2012–2021 (n = 105)

**Figure 2:**
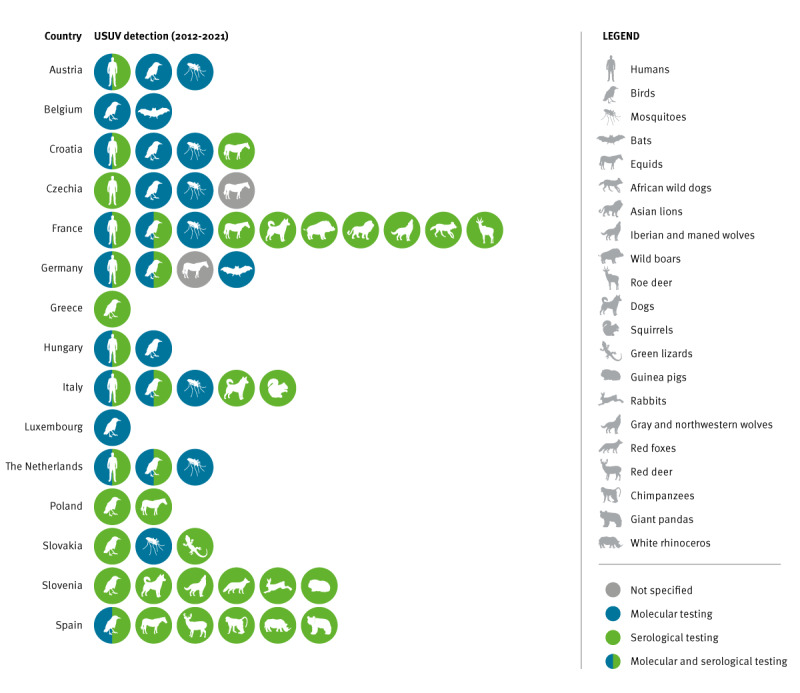
Usutu virus infections in humans and in animals, EU/EEA countries, 2012–2021

Based on this compiled information, during the period 2012 to 2021, 105 autochthonous cases of USUV infection were diagnosed in humans in Austria, Croatia, Czechia, France, Germany, Hungary, Italy and the Netherlands (Figure 1 and Figure 2). Most cases occurred in Italy (n = 56), Austria (n = 27) and the Netherlands (n = 11). Reports on USUV infection in humans have almost doubled (n = 70) from 2017 to 2021, mainly due to the upsurge of cases in 2018, in line with the increase of human cases of WNV [15,18,19]. Cases of USUV infection were identified in retrospective studies of persons with neuroinvasive diseases [14], in surveys of blood donors [7,8], via routine diagnostics in patients with neurological symptoms or unexplained fever [13,15,16,19] and by differential diagnosis by NAT screening of blood donors testing positive for WNV [3,6,15,17,18,20]. Twelve cases from Austria, Croatia, Czechia, France, Hungary and Italy had neurological symptoms, eight Italian cases had a febrile illness and the remaining 85 cases were asymptomatic individuals, mainly blood donors. None of the cases with USUV neuroinvasive disease was immunocompromised.

EU/EEA: European Union/European Economic Area.

EU/EEA: European Union/European Economic Area; USUV: Usutu virus.

Blue colour indicates detection by molecular methods, green colour indicates detection by serology, grey colour indicates not specified method for detection.

#### Infections in animals

Fifteen EU countries (Austria, Belgium, Croatia, Czechia, France, Germany, Greece, Hungary, Italy, Luxembourg, the Netherlands, Poland, Slovakia, Slovenia and Spain) reported USUV circulation among vertebrate animals, mostly in birds by detection of the virus and/or anti-USUV antibodies and occasionally in equids using serology (Figure 2) [3,4,10,21-30]. According to our survey, all countries reporting human cases had also evidence of USUV circulation among birds and/or mosquitoes (Figure 2). Usutu virus was detected in 11 countries in resident or captive wild birds and in 10 countries in migratory birds. Specifically, the most affected wild migratory and/or resident bird species (sp.) were Passeriformes (mainly *Turdus* sp., particularly Eurasian blackbird, barn swallow, European goldfinch), Accipitriformes (common buzzard, hawk, goshawk), Strigiformes (owls) and Columbiformes (common wood pigeons, collar dove) [28,31-33]. Infection with USUV may cause outbreaks and lead to high mortality in captive zoo birds that thus can be useful sentinels for early detection of the virus circulation. Surveys among zoo birds demonstrated high seroprevalence of USUV in common peafowls, emus and greater rheas in France [34]; antibodies against USUV were detected in pelicans, Eurasian eagle owls, barn and snowy owls in Slovenia [35] and in a greater rhea in Spain [36]. In Germany, a nationwide bird surveillance network has been established to monitor zoonotic arbovirus infections (with focus on USUV and WNV) in migratory and resident wild birds, including captive birds [28,31]. Via this surveillance activity, between 2019 and 2020, USUV RNA was detected in 1.2–2.7% of live wild birds and in 2.8–12.2% of dead birds from all regions of the country. Among captive birds, USUV RNA was detected in several owls, in Harris’s hawk, steppe eagle, Eurasian capercaillie, black-tailed gull, red-breasted goose and in a *Penguin* sp [28]. In seven EU/EEA countries, USUV was detected in other animals than birds and equids, i.e. in pets, wild and zoo animals (details in Figure 2).

Usutu virus was detected in mosquitoes in Austria, Croatia, Czechia, France, Italy, the Netherlands and Slovakia [2,10,24,37-39]. In these countries, the virus was mostly detected in *Culex pipiens* (Austria, Croatia, Czechia, France, Germany, Italy, Slovakia), but also in *Cx. modestus* (Czechia, Slovakia), *Aedes albopictus* (Italy), *Ae. japonicus* (Austria), *Anopheles hyrcanus* (Slovakia), *An. maculipennis* s.l. (Slovakia) and *Ochlerotatus caspius* (Italy).

### Surveillance

#### Human surveillance

We collected information on USUV surveillance activities implemented in the EU/EEA. The following countries responded to the survey on USUV surveillance in humans: Austria, Belgium, Croatia, Czechia, Estonia, Finland, France, Germany, Greece, Hungary, Iceland, Ireland, Italy, Latvia, Liechtenstein, Lithuania, Luxembourg, Malta, the Netherlands, Norway, Romania, Slovakia, Slovenia and Spain. According to the survey, USUV infection in humans, whether symptomatic or asymptomatic, was only notifiable in Italy. In particular, since 2017, Italy implemented a joint USUV and WNV surveillance integrated in a One Health approach, i.e. targeting humans, birds, equids and mosquitoes [40]. The goal of the USUV surveillance in Italy is to identify areas at high risk of human disease and to activate control measures [40]. In Greece and Norway, USUV infection is not specifically mentioned as notifiable but is included in the notifiable category “arboviral encephalitis” and “viral infections of the central nervous system”, respectively. Although not within national surveillance, some EU/EEA countries routinely include USUV testing in patients suspected of viral meningitis or encephalitis (Croatia, Germany, Greece and Norway) and in the differential diagnosis for confirmation of probable WNV cases and WNV NAT-positive blood donors (Austria, Croatia, Czechia, France, Germany, Greece, Hungary, Latvia, Liechtenstein, the Netherlands, Slovenia and Spain). In Germany, Greece, Italy and Norway national case definitions for USUV infection in humans have been developed, described in the Box.

Box **Case definition of Usutu virus infection and laboratory criteria for confirmation in humans**

**Italy:**

• A case of USUV infection is confirmed in individuals with or without symptoms, by virus isolation and/or nucleic acid detection from any clinical specimen, detection of USUV-specific IgM antibodies in CSF, detection of USUV IgM antibodies at high titre and IgG antibodies in serum, confirmed by a neutralisation assay. Any person with fever or neurological manifestations (encephalitis, meningitis with clear CSF, polyradiculoneuritis, or acute flaccid paralysis) and USUV-specific IgM response in serum, without any confirmation by neutralisation assay, is classified as a probable USUV case.

**Germany:**

• A case of USUV infection is defined by detection of USUV RNA or USUV-specific antibodies, confirmed by neutralisation assay.

**Greece:**

• A case of USUV infection is defined by virus isolation and/or nucleic acid detection from any clinical specimen, demonstration of increased titre of antibodies in serum, or detection of USUV-specific IgM antibodies in CSF.

**Norway:**

• Criteria for notification are laboratory detection of USUV in CSF by isolation or nucleic acid detection or detection of specific antibody response in serum and/or CSF.

CSF: cerebrospinal fluid; USUV: Usutu virus.

#### Animal surveillance

The following countries responded to the survey on USUV surveillance in animals: Austria, Croatia, Cyprus, Czechia, Denmark, Estonia, Finland, France, Germany, Greece, Hungary, Ireland, Italy, Latvia, Lithuania, Luxembourg, the Netherlands, Norway, Poland, Portugal, Romania, Slovakia, Slovenia and Spain. Results showed that, in animals, USUV infection is not notifiable at a national level in any EU/EEA country. Surveillance of USUV in animals has been implemented in seven EU countries (Denmark, France, Germany, Hungary, Italy, Luxembourg and Spain), while in Finland and the Netherlands, USUV surveys have been carried out through specific research projects.

The target species of USUV surveillance in animals vary between countries. In Denmark, Germany and Luxembourg only birds are included whereas both birds and other animals (i.e. wild boars, roe deer, cattle and equids) are included in Italy and France. In addition, there are differences among the bird species investigated: only wild birds in France and Luxembourg and both wild and domestic birds in Denmark, Germany and Italy.

Only Italy has developed a national case definition for USUV infection in animals, which includes detection of USUV IgM and IgG antibodies in serum by ELISA, confirmed by a serum neutralisation assay. The case definition also covers detection of USUV RNA in mosquito pools or organs or blood samples of birds, either captured or found dead, by the local animal health units, which are the competent authority and confirmed by national reference laboratories.

Ten EU countries collect data on USUV and WNV in mosquitoes. In seven countries, i.e. Austria, Croatia, Denmark, Germany, Greece, Italy and the Netherlands, data on USUV infection in mosquitoes are collected through national surveillance activities. Three countries, i.e. France, Slovakia and Slovenia, collect data on WNV/USUV infection in mosquitoes through recurring or occasional research projects carried out by universities and national research institutes.

#### Diagnostic capability and diagnosis

Twenty EU/EEA countries (Austria, Belgium, Croatia, Czechia, Estonia, France, Germany, Greece, Hungary, Ireland, Italy, Latvia, Liechtenstein, Lithuania, Malta, the Netherlands, Romania, Slovakia, Slovenia, Spain) have the laboratory capability to diagnose USUV infection in humans, mostly by the national reference laboratories. The differential diagnosis between WNV and other flavivirus infections, including USUV, is part of routine practice in WNV NAT-positive SoHO donors in seven countries (i.e. Austria, Czechia, France, Germany, Italy, the Netherlands and Slovakia). The laboratory tests used in the EU/EEA countries to detect USUV include USUV-specific PCR or broad-range pan-flavivirus PCR and sequencing and viral isolation in cell culture from blood, urine, CSF and any other patient samples. Serological diagnosis relies on the detection of USUV-specific antibody response by immunoassay or the detection of flavivirus antibody response using cross-reactive serological methods. Both serological tests require confirmation by neutralisation assay.

Similarly, the laboratory tests used in 20 EU/EEA countries (Austria, Croatia, Cyprus, Czechia, Denmark, France, Germany, Greece, Hungary, Ireland, Italy, Latvia, Lithuania, Luxembourg, the Netherlands, Poland, Portugal, Slovakia, Slovenia and Spain) to identify USUV infection in animals include USUV-specific PCR or broad-range pan-flavivirus PCR and sequencing from blood, tissue and/or CSF. Serological diagnosis can be performed through the detection of USUV specific antibody response or the flavivirus antibody response using other cross-reactive serological methods (e.g. haemagglutination-inhibition, indirect immunofluorescence), followed by a neutralisation assay.

#### Safety of substances of human origin

None of the EU/EEA countries in our study had implemented USUV-specific SoHO safety measures to prevent USUV transmission via SoHO donations. However, countries performing WNV NAT screening of SoHO donors can also detect USUV infection and thus exclude USUV-positive donations from transfusions or transplantation. So far, no donor-derived USUV infections have been reported.

## Discussion

According to our study, between 2012 and 2021, half of the EU/EEA countries reported circulation of USUV in humans and animals. Outside the EU/EEA, USUV circulation was reported in the UK [41,42], Switzerland [43] and Serbia [44], during the same period. In addition, since the grey literature was not included in our search, we cannot exclude that the other European countries reported USUV circulation.

These results may pose questions about the impact of USUV infection on human health, including the potential risk of virus transmission through SoHO donations. However, the incidence of USUV infection in humans cannot be easily determined, since most infections are asymptomatic or with mild and nonspecific symptoms, while central nervous system involvement is rare. Moreover, in most EU/EEA countries, USUV infection in humans is not under surveillance and USUV testing is not routinely performed in patients with neurological symptoms or suspected arbovirus infection. Therefore, the diagnosed USUV human infections conceivably represent only the tip of the iceberg. Retrospective serological studies of healthy individuals and WNV NAT screenings of blood donors revealed that humans might be exposed to USUV infection more than expected and, in some circumstances, the likelihood of USUV infection might be even higher than of WNV infection, as described in studies and reports from Austria, France, Italy and the Netherlands [8-10,18]. On the other hand, USUV is less pathogenic for humans than WNV, as indicated by the markedly fewer reported neuroinvasive infections caused by USUV than WNV [10]. In Italy, where both USUV and WNV are, since 2017, included in a joint surveillance programme, the number of reported cases of WNV neuroinvasive infections and the ratio of WNV neuroinvasive cases of all diagnosed WNV cases, have been markedly higher than the USUV-related cases [15]. Low pathogenicity of USUV was also suggested in a retrospective investigation of three recipients of erythrocytes and platelets from USUV-positive donors, as none developed symptoms or seroconverted [8]. However, lack of seroconversion or asymptomatic infections have been also reported in some SoHO recipients of WNV-positive donors, although other SoHO recipients of the same donors developed encephalitis [45,46].

Experimental studies in vitro and in animal models confirmed USUV had lower virulence than WNV, but USUV was able to invade the brain and replicate in neural cells [47-50]. However, since pathogenicity may vary among USUV strains, as shown in experimental mice models [51], close monitoring of USUV incursions and evolution by full genome sequencing and studies in experimental models to determine genotype-phenotype correlates of pathogenicity are warranted.

Knowledge of USUV ecology, including reservoir species, competent vectors, interaction with WNV and the impact of climate factors on viral transmission cycle would also be crucial to estimate the epidemic potential of the virus. At least 58 bird species from 13 orders have been found infected with USUV in continental Europe [28,31-36]. Recognised reservoirs (i.e. species that are able to maintain, or significantly contribute to maintaining the virus in nature or contribute to the virus circulation) and amplifying hosts (i.e. a host in which the virus multiplies rapidly to high levels, providing an important source of infection for vectors) of USUV are especially in the orders Passeriformes and Strigiformes. The available epidemiological data provide little information on the potential role of many other bird species as reservoirs and/or amplifying hosts of the virus, as well as on the relevance in USUV life cycle of other mammal species in European countries. It is important to correctly target the animal species with higher prevalence of the infection in the surveillance programmes. The target species may vary between countries.

The geographical distribution and ecological cycle of USUV largely overlap that of WNV, but few studies have tried to understand how co-infections or subsequent infections within the same host or vector affect the immune response and susceptibility to subsequent infection or vector competence. Virus co-circulation also impacts diagnostics because of the difficulties to distinguish between the two viruses. Finally, virus co-circulation might affect virus evolution and geographical distribution under host selection pressures or competition between viruses. Experimental data indicate that previous exposure to USUV partially protects magpies against lethal challenge with WNV but does not prevent viraemia and possible direct transmission [52]. However, another study of experimental infection in geese showed that prior USUV infection protected the birds from clinical disease and led to significantly lower viraemia in a subsequent WNV infection [53]. In line with these results, field surveillance of migratory and resident birds in Germany identified WNV and USUV co-infection in six dead birds [54]. Further investigation is needed to clarify if USUV circulation among birds affects WNV transmission cycle and vice versa. In experimental infection of *Culex* mosquitoes, pre-infection with USUV significantly reduced WNV transmission [55]. In mice, prior immunity against WNV protected against subsequent USUV clinical illness [56] and vice versa [50].

*Culex pipiens* is considered the most common vector for USUV in EU/EEA countries although some other mosquito species, i.e. *Cx. modestus*, *Cx. torrentium* [57], *Ae. albopictus* [58] and *Ae. japonicus* [59], have been demonstrated to be potential vectors in laboratory studies. In nature, USUV has been detected in many other mosquito species, such as *Cx. perexiguus*, *Anopheles maculipennis* sensu lato, *Culiseta annulata*, *Ochlerotatus caspius* and *Oc. detritus*. However, their capability to maintain and transmit USUV needs to be demonstrated.

Analysis of data from peer-reviewed literature and the survey highlighted an increased scientific interest in USUV as an emerging pathogen and awareness about the risk of disease in humans. Several countries in the EU/EEA are investigating USUV circulation in animals and mosquitoes, mainly in the context of WNV surveillance and research and have included USUV testing in the differential diagnosis of patients with probable WNV infection. Some countries (Box) have developed a case definition for USUV infection in humans and animals and Italy also included it among notifiable diseases. Most EU/EEA countries have the laboratory capability for USUV testing in humans and animals, although generally limited to the reference laboratories.

## Conclusion

In the past 10 years, at least half of the EU/EEA countries have found evidence of USUV infection in humans or animals. Most USUV infections in humans were asymptomatic and rarely associated with neuroinvasive disease and the impact of USUV on public health was much lower than for WNV. However, this could be because of lack of testing for USUV or not including USUV in the differential diagnosis of probable WNV cases. Further research will be required to better understand the epidemiology and the ecology of the virus and the impact of WNV and USUV co-circulation. To date USUV does not pose a major public health threat to the EU, but monitoring virus circulation and pathogenicity is important to early detect any change in the epidemiology of the disease.

## Supplementary Data

150000 Supplementary Material
