# Author’s correction for Euro Surveill. 2023;28(33)

**DOI:** 10.2807/1560-7917.ES.2023.28.34.230824c

**Published:** 2023-08-24

**Authors:** Eurosurveillance editorial team

**Affiliations:** 1 European Centre for Disease Prevention and Control (ECDC), Stockholm, Sweden

In the article entitled ‘Epidemiology, surveillance and diagnosis of Usutu virus infection in the EU/EEA, 2012 to 2021’ by Angeloni et al. the number of cases with neurological symptoms was incorrect in the abstract (11 cases). This mistake was corrected at the request of the authors on 22 August 2023 (the correct number is 12).

