# Supplementary material for: Epidemiology, surveillance and diagnosis of Usutu virus infection in the EU/EEA, 2012 to 2021
Source: Euro Surveill. 2023 Aug 17;28(33):2200929. doi: 10.2807/1560-7917.ES.2023.28.33.2200929 (PMC10436690; doi:10.2807/1560-7917.ES.2023.28.33.2200929)
Supplement: Supplementary Material [file 22-00929_BARZON_Supplement.pdf]

Supplementary Material

This supplementary material is hosted by *Eurosurveillance* as supporting information alongside the article **Epidemiology, surveillance, and diagnosis of Usutu virus infection in the EU/EEA, 2012 to 2021**, on behalf of the authors, who remain responsible for the accuracy and appropriateness of the content. The same standards for ethics, copyright, attributions and permissions as for the article apply. Supplements are not edited by *Eurosurveillance* and the journal is not responsible for the maintenance of any links or email addresses provided therein.

Methods

Data and information on USUV epidemiology, surveillance and diagnosis in 30 EU/EEA during the period 2012 to 2021 were collected through:

(i) a review of peer-reviewed articles reporting USUV detection in humans, vertebrate animals and mosquitoes from 2012 to 2021 using five search engines and platforms (PubMed, Web of Science, Scopus, Embase and CAB-abstracts). Research strings used to retrieve peer-reviewed articles from the literature are reported in the Supplementary Table. Two reviewers (MB, EL) categorized all studies obtained via the initial literature search based on title and abstract. In the case of a poorly explicative abstract or in the case of doubt about the available data, the study was included and evaluated at full-text level. Each record was coded twice, i.e., separately by two reviewers, and a third reviewer solved conflicts (AG). After full texts retrieval, two reviewers (MB, EL) extracted data from included studies and were entered into pre-defined tabular forms. An independent cross-check control on the extracted data with the original data in the studies was perform by the two reviewers.

Supplementary Table. Research strings used in the review of the literature.

Database	Query USUV
PubMed	("usutu"[Title/Abstract] AND ("Austria"[Title/Abstract] OR "Belgium"[Title/Abstract] OR "Bulgaria"[Title/Abstract] OR "Croatia"[Title/Abstract] OR "czech*"[Title/Abstract] OR "Denmark"[Title/Abstract] OR "Estonia"[Title/Abstract] OR "Finland"[Title/Abstract] OR "France"[Title/Abstract] OR "Germany"[Title/Abstract] OR "Greece"[Title/Abstract] OR "Hungary"[Title/Abstract] OR "Iceland"[Title/Abstract] OR "Ireland"[Title/Abstract] OR "Italy"[Title/Abstract] OR "Latvia"[Title/Abstract] OR "Liechtenstein"[Title/Abstract] OR "Lithuania"[Title/Abstract] OR "Luxembourg"[Title/Abstract] OR "Malta"[Title/Abstract] OR "Netherlands"[Title/Abstract] OR "Norway"[Title/Abstract] OR "Poland"[Title/Abstract] OR "Portugal"[Title/Abstract] OR "Cyprus"[Title/Abstract] OR "Romania"[Title/Abstract] OR "Slovakia"[Title/Abstract] OR "Slovenia"[Title/Abstract] OR "Spain"[Title/Abstract] OR "Sweden"[Title/Abstract] OR "europe*"[Title/Abstract]))
Web of Science	(TS=(usutu) AND TS=(Austria OR Belgium OR Bulgaria OR Croatia OR Czech OR Denmark OR Estonia OR Finland OR France OR Germany OR Greece OR Hungary OR Iceland OR Ireland OR Italy OR Latvia OR Liechtenstein OR Lithuania OR Luxembourg OR Malta OR Netherlands OR Norway OR Poland OR Portugal OR Cyprus OR Romania OR Slovakia OR Slovenia OR Spain OR Sweden OR Europe))
Scopus	(TITLE-ABS-KEY (austria OR belgium OR bulgaria OR croatia OR czech OR denmark OR estonia OR finland OR france OR germany OR greece OR hungary OR iceland OR ireland OR italy OR latvia OR liechtenstein OR lithuania OR luxembourg OR malta OR netherlands OR norway OR poland OR portugal OR cyprus OR romania OR slovakia OR slovenia OR spain OR sweden OR europe)) AND (TITLE-ABS-KEY (usutu))
Embase	'usutu':ab,ti AND ('austria':ab,ti OR 'belgium':ab,ti OR 'bulgaria':ab,ti OR 'croatia':ab,ti OR 'cyprus':ab,ti OR 'czech republic':ab,ti OR 'denmark':ab,ti OR 'estonia':ab,ti OR 'finland':ab,ti OR 'france':ab,ti OR 'germany':ab,ti OR 'greece':ab,ti OR 'hungary':ab,ti OR 'iceland':ab,ti OR 'ireland':ab,ti OR 'italy':ab,ti OR 'latvia':ab,ti OR 'liechtenstein':ab,ti OR 'lithuania':ab,ti OR 'luxembourg':ab,ti OR 'malta':ab,ti OR 'netherlands':ab,ti OR 'norway':ab,ti OR 'poland':ab,ti OR 'portugal':ab,ti OR 'romania':ab,ti OR 'slovakia':ab,ti OR 'slovenia':ab,ti OR 'spain':ab,ti OR 'sweden':ab,ti OR 'europe':ab,ti)
CAB abstracts	((ab:(usutu) AND ab:((Austria) OR (Belgium) OR (Bulgaria) OR (Croatia) OR (Czech) OR (Denmark) OR (Estonia) OR (Finland) OR (France) OR (Germany) OR (Greece) OR (Hungary) OR (Iceland) OR (Ireland) OR (Italy) OR (Latvia) OR (Liechtenstein) OR (Lithuania) OR (Luxembourg) OR (Malta) OR (Netherlands) OR (Norway) OR (Poland) OR (Portugal) OR (Cyprus) OR (Romania) OR (Slovakia) OR (Slovenia) OR (Spain) OR (Sweden) OR (Europe)))

(ii) an online survey distributed to the European Centre for Disease Prevention and Control (ECDC) National

Focal Points (NFP) for Emerging and Vector-borne Diseases (EVDs), European Food Safety Authority (EFSA) NFP and SoHO national competent authorities in the period from July to September 2021. The ECDC NFPs are official representatives of the national public health institutes and the EFSA NFPs are official representatives of the food safety and/or the veterinary authorities or institutes in the EU/EEA countries. EFSA NFPs requested to forward the survey to the National Competent Veterinary Authority. Before launching the survey, the questionnaire was tested by three public health experts and one animal health expert, in order to identify and eliminate any unclear or dubious questions and to adjust content and formatting. Three reminders were sent during the survey collection period. Twenty-nine countries out of 30 provided a reply for at least one sector. In 10 countries, two respondents completed the same section of the questionnaire. In nine of these cases, since some contradictory answers were present, respondents from the same country were asked to revise their answers and to agree on a common version (in September 2021). Finally, 66 respondents were considered in the analysis.

(iii) a technical stakeholder meeting involving representatives from national veterinary and public health institutes and SoHO authorities in addition to invited scientific experts. The aim of the meeting was to share and clarify the information collected though the questionnaire. The online technical stakeholder meeting was organised on 18 and 19 January, 2022 (half-day). Representatives from national veterinary, public health, and SoHO institutes and/or ministries from 13 EU/EEA countries (Austria, Croatia, Cyprus, France, Germany, Greece, Hungary, Italy, the Netherlands, Portugal, Romania, Slovenia and Spain) were invited. In addition, representatives from ECDC/EFSA funded networks (VectorNet covering the entomological aspects and EVD-LabNet covering the diagnostic laboratory aspects) and the EU Commission attended the meeting. Overall, 77 and 69 participants attended in the first and the second day meetings, respectively. Controversial results that were collected through the survey were clarified during the meeting and properly corrected. The main points to be clarified were addressed through specific talks and presentations to facilitate the discussion.

Although UK was an EU/EEA country until the end of 2020, this country was not included in the online survey and the technical stakeholder meeting, since they were done in 2021-2022.
